# Transsphenoidal Surgery of Corticotroph Adenomas With Cavernous Sinus Invasion: Results in a Series of 86 Consecutive Patients

**DOI:** 10.3389/fonc.2022.810234

**Published:** 2022-02-08

**Authors:** Congxin Dai, Ming Feng, Lin Lu, Bowen Sun, Yanghua Fan, Xinjie Bao, Yong Yao, Kan Deng, Renzhi Wang, Jun Kang

**Affiliations:** ^1^ Department of Neurosurgery, Beijing Tongren Hospital, Capital Medical University, Beijing, China; ^2^ Department of Neurosurgery, Peking Union Medical College Hospital, Chinese Academy of Medical Sciences and Peking Union Medical College, Beijing, China; ^3^ Department of Endocrinology, Peking Union Medical College Hospital, Chinese Academy of Medical Sciences and Peking Union Medical College, Beijing, China

**Keywords:** corticotroph adenomas, cavernous sinus invasion, transsphenoidal surgery, remission, recurrence

## Abstract

**Objective:**

Transsphenoidal surgery (TSS) is the first-line treatment for corticotroph adenomas. Although most corticotroph adenomas are noninvasive microadenomas, a small subset of them invading cavernous sinus (CS) is notoriously difficult to manage. The aim of this study was to evaluate the surgical outcome of corticotroph adenomas with CSI from a single center.

**Patients and Methods:**

The clinical features and outcomes of CD patients who underwent TSS between January 2000 and September 2019 at Peking Union Medical College Hospital were collected from medical records. The clinical, endocrinological, radiological, histopathological, and surgical outcomes, and a minimum 12-month follow-up of patients with corticotroph adenomas invading CS were retrospectively reviewed.

**Results:**

Eighty-six patients with corticotroph adenomas invading CS were included in the study. The average age at TSS was 37.7 years (range, 12 to 67 years), with a female-to-male ratio of 3.1:1 (65/21). The median duration of symptoms was 52.6 months (range, 1.0 to 264 months). The average of maximum diameter of tumor was 17.6 mm (range, 4.5–70 mm). All included 86 patients underwent TSS using a microscopic or an endoscopic approach. Gross total resection was achieved in 63 patients (73.3%), subtotal resection was attained in 18 (20.9%), and partial resection was achieved in 5 (5.8%). After surgery, the overall postoperative immediate remission rate was 48.8% (42/86); 51.2% (44/86) of patients maintained persistent hypercortisolism. In 42 patients with initial remission, 16.7% (7/42) experienced a recurrence. In these patients with persistent disease and recurrent CD, data about further treatment were available for 30 patients. Radiotherapy was used for 15 patients, and 4 (26.7%) of them achieved biochemical remission. Repeat TSS was performed in 5 patients, and none achieved remission. Medication was administered in 4 patients, and one of them obtained disease control. Adrenalectomy was performed in 6 patients, and 5 (83.3%) achieved biochemical remission. At the last follow-up, 10 of 30 patients (33.3%) were in remission, and 20 patients still had persistent disease.

The remission rate in corticotroph adenomas with cavernous sinus invasion (CSI) that underwent gross total resection and first TSS was significantly higher than that in patients undergoing subtotal resection, partial resection, and a second TSS (all p < 0.05). However, there was no significant difference in the remission rate between patients with different tumor sizes, Knosp grades, and surgical approaches (p > 0.05).

**Conclusion:**

The management of corticotroph adenomas with CSI remain a therapeutic challenge due to incomplete resection of invasive and/or a large adenoma. With the application of multiple techniques, approximately half of the patients could achieve gross total resection and biochemical remission *via* TSS by experienced neurosurgeons. The extent of tumor resection and the number of operations were associated with surgical remission rate in corticotroph adenomas with CSI. If the remission was not achieved by surgery, other treatments including radiotherapy, medical therapy, and even bilateral adrenalectomy are required.

## Introduction

Cushing disease (CD) is defined as chronic hypercortisolemia, which is mainly caused by corticotroph adenomas. CD is a relatively rare disease with an incidence of approximately 0.7–2.4 cases per million persons/year (1). If untreated or incompletely controlled, CD is associated with increased morbidity and higher mortality rate compared to the general population (2). Transsphenoidal surgery (TSS) is considered the first-line treatment for patients with CD. However, the remission rate after TSS has been reported to vary widely from 59% to 94% (3). Most corticotroph adenomas are non-invasive microadenomas with a tumor diameter of less than 8 mm (4, 5). The surgical remission rates in these patients with noninvasive visible microadenomas frequently exceed greater than 80% (6). However, surgery for corticotroph adenomas with cavernous sinus invasion (CSI) has represented challenge, and the remission rates tended to be extremely low. Because corticotroph adenomas with CSI are relatively rare, there is still no comprehensive study on large-scale cases of corticotroph adenomas with CSI. To date, surgical outcome and perioperative complications of corticotroph adenomas with CSI have not been clearly reported (7). The purpose of this study was to assess the surgical outcome and perioperative complications of corticotroph adenomas with CSI.

## Patients and Methods

We conducted a retrospective analysis of consecutive patients with corticotroph adenoma invading CS who underwent TSS at Peking Union Medical College Hospital (PUMCH) between January 2000 and September 2019. According to the guidelines of the PUMCH Medical Ethical Committee, clinical information on age, sex, clinical manifestation, preoperative biochemical parameters and imaging results, operative findings, postoperative hormone levels and pathology, long-term outcomes, and follow-up were collected for analysis.

### Inclusion and Exclusion Criteria

The inclusion criteria for this study were as follows: (1) the CD was diagnosed based on the patient’s history and results of a physical examination, laboratory tests, magnetic resonance imaging (MRI), and surgical findings; (2) a corticotroph adenomas was confirmed on immunohistopathological studies; (3) the CSI was recognized by both preoperative MRI results and intraoperative findings; and (4) with complete medical records and a minimum of 2 years of follow-up. According to the Knosp grade classification (8), if tumors were classified as Knosp grade of 2 based on preoperative MRI results, but the CSI was confirmed intraoperatively, these patients were also included in this study. If the tumors were classified as Knosp grade of 3 according to the preoperative MRI results, but no CSI was recognized intraoperatively, these patients were excluded from the study. Patients were excluded if the surgery was aborted because of carotid artery bleeding, surgery was *via* a transcranial approach, the medical records were not complete, or lack of pathological report or follow-up data.

### Diagnosis of Corticotroph Adenomas with CSI

Most patients were diagnosed as having corticotroph adenomas with CSI by a pituitary multidisciplinary team (MDT) including members from the Departments of Neurosurgery, Endocrinology, Radiology, and Pathology, as we described in a previous study (9). All corticotroph adenomas with CSI were diagnosed based on clinical manifestation, endocrine examinations, radiological examinations, intraoperative observation, and pathological results. In our center, all CD patients underwent comprehensive biochemical tests, including morning serum cortisol and ACTH, 24-h urine-free cortisol (UFC), and a combined low-dose and high-dose dexamethasone suppression test (LDDST and HDDST). The methods of LDDST and HDDST have been described in detail in our previous study (10). If 24-h UFC and cortisol were not suppressed on the LDDST but was suppressed on the HDDST, CD was diagnosed. Based on our previous research results, the sensitivity of combined LDDST and HDDST to diagnose CD is 88.53% in our center (11).

Preoperatively, all patients underwent pituitary contrast-enhanced MRI and/or dynamic gadolinium-enhanced MRI. Images were independently reviewed by one neuroradiologist and at least one neurosurgeon, to evaluate the invasiveness of tumors, and ensure that agreement was achieved on Knosp grade, which was described previously (8). Adenomas of Knosp Grade 0–2 were defined as non-invasive PAs, and adenomas of Knosp Grade 3–4 were defined as invasive PAs, respectively. However, preoperative diagnosis of CSI by PAs based on MRI has always been inconsistent with intraoperative exploratory results. With the advancement of endoscopic techniques, the medial wall of the CS can be exposed more clearly and directly observed. Therefore, whether a PA presents CSI was finally confirmed by preoperative Knosp grading combined with intraoperative observed results. According to the maximal diameter, adenomas were categorized as microadenoma (<1 cm), macroadenoma (<4 cm), and giant adenoma (≥4 cm).

### Surgical Procedures

All TSSs were performed in the Department of Neurosurgery in PUMCH. Almost all the included patients were operated on by the same two experienced surgeons (RW and MF). The adenomas were removed via a transsphenoidal microscopic or an endoscopic approach, or a combination of them. The extended transsphenoidal approach assisted by multiple techniques was performed in some invasive macroadenomas or giant adenomas as previously described (12). In surgery, standard transsphenoidal PAs resection via a microscopic approach or an endoscopic approach was performed after broadly opening the sella floor. The tumor localized in the intrasellar and suprasellar region was removed first and then the residual tumor in the cavernous sinus (CS) was inspected. The medial wall of the CS was explored under direct vision, and soft tumor tissue was removed using suction and ring curettes. The ICA was located using intraoperative mini-Doppler. The anterior wall of the CS was opened carefully and then the soft tumors located lateral to the ICA was removed by suction and ring curettes. If the tumor was exposed insufficiently, the ICA could be gently pushed to increase visualization for further exposure. If the tumor was too hard to remove, or the tumor firmly adhered to the ICA or nerves, leave the tumor to avoid injuring the ICA or nerves. The residual tumors will be addressed via postoperative radiotherapy. Finally, the sella was reconstructed in all patients to avoid postoperative cerebrospinal fluid (CSF) leakage and CNS infection.

### Classifications of Intraoperative Observed CSI

The CSI was determined by the experienced neurosurgeons during surgery based on the degree of invasion of the adenoma into the CS wall and the presence or absence of adenomas within the CS. After tumor removal, if the medial wall of CS was intact and smooth, absence of invasion was considered. If the medial wall was not intact, intracavernous ligaments, ICA, or nerve fibers were visible, and CSI was considered.

### Extent of Tumor Resection

The extent of tumor resection was evaluated through MRI examinations within 3 days and on 3 months after initial surgery. The gross total resection (GTR) was defined as no residual tumor was identified. The subtotal resection (STR) was defined as more than 90% volume compared with when the preoperative volume was removed, or when a definite residual tumor volume less than 10% compared with when the preoperative volume was identified. Partial resection was defined as residual tumor volume greater than 10% (13).

### Histological Analysis

The resected specimens of corticotroph adenomas were performed using routine H & E histological stains and immunohistochemical tests for ACTH. The diagnosis of CD was confirmed if pathology under light microscopy demonstrated an adenoma with immunohistological staining positive for ACTH.

### Surgical Outcome Measures

After TSS, the endocrinological tests were performed within the first 7 days, 1 month, 3 months, and 12 months, respectively. As we described previously (14), immediate remission was defined as a serum cortisol level <5 µg/dl, and/or 24-h UFC level fell below 20 µg (56 nmol) within the first 7 days after TSS. Persistent disease was defined in patients as elevated postoperative cortisol levels and a need for additional therapy. Recurrence was defined in patients who achieved initial remission, followed by a rise in serum cortisol or 24-h UFC and associated with clinical symptoms of CD. To assess the degree of tumor resection, postoperative MRI was done within 3 days and on 3 months after the surgery.

### Statistical Analysis

Statistical significance for continuous variables was determined using the Student’s *t*-test or analysis of variance. Categorical variables were analyzed using either the Pearson *χ* ^2^ test or Fisher exact test. Pearson *χ* ^2^ testing for determination of statistical significance was performed by using either a 2 · 2 contingency table or test for independence if greater than 2 outcomes were being analyzed. Fisher exact test was used if any expected values were less than or equal to 5. Statistical significance was defined as a *p*-value less than.05. All statistical analyses were completed by using SPSS software, version 22 (SPSS, Inc., Chicago, Illinois).

## Results

### Demographic and Clinical Characteristics of Included Patients

From January 2000 to September 2019, 1,381 patients with CD had TSS at the PUMCH, and the intraoperatively observed corticotroph adenomas invading CS accounted for 7.5% (104/1,381) of overall patients. Of these, 86 patients with complete medical records, pathological confirmation, and a minimum 2-year follow-up were included in this study. The detailed information is listed in **Table 1**. There are 21 male patients and 65 female patients, and the median duration of symptoms was 52.6 months (range, 1.0 to 264 months). The average age at TSS was 37.7 years (range, 12 to 67 years), and the mean follow-up time was 49.5 months (range, 24.0–159.0 months).

**Table 1:** Demographic and clinical characteristics of 86 patients with corticotroph adenomas invading cavernous sinus.

Characteristics	Value
**Total**	86
**Gender**	
Female (*n*, %)	65 (75.6%)
Male (*n*, %)	21 (24.4%)
**Mean age, years (range)**	37.7 (12–67)
**Number of operations**	
First (*n*, %)	66 (76.7%)
Repeat (*n*, %)	20 (23.3%)
**Disease duration, months (range)**	52.6 (1–264)
**BMI**	26.6 (18.9–36.7)
**Tumor size (mean) (range)**	17.6 mm (4.5–70.0 mm)
Microadenoma (*n*, %), Diameter (mm)	(28, 32.6%) 6.9 mm
Macroadenoma (*n*, %), Diameter (mm)	(54, 62.8%) 20.0 mm
Giant adenoma (*n*, %), Diameter (mm)	(4, 4.7%) 60.6 mm
**Preoperative 24-h UFC level (μg)**	721.4 (71.3–3705.6)
**Preoperative serum cortisol level (μg/dl)**	28.9 (7.4–75.0)
**Preoperative ACTH level (ng/L)**	142.5 (23.1–1250.0)
**Combined LDDST and HDDST test**	
Positive (*n*, %)	72 (83.7%)
Negative (*n*, %)	14 (16.3)
**Follow-up time, months (range)**	49.5 (24.0–159.0)

The median tumor diameter of overall patients was 17.6 mm (range 4.5–70.0 mm). Of these, 32.6% (28/86) of patients demonstrated microadenomas with a median tumor diameter of 6.9 mm, 62.8% (54/86) of patients demonstrated macroadenomas with a median tumor diameter of 20.0 mm, and 4.7% (4/86) of patients had giant adenomas with a median tumor diameter of 60.6 mm, respectively. Mean preoperative body mass index (BMI) was 26.6 (range, 18.9–36.7 kg/m^2^). The median preoperative 24-h UFC levels, morning serum cortisol, and ACTH were 721.4 μg (range, 71.3–3,705.6), 28.9 μg/dl (range, 7.4–75.0), and 142.5 ng/L (range, 23.1–1,250.0), respectively. In these cases, 66 patients underwent first TSS, and 20 patients underwent repeated TSS, respectively. In 83.7% (72/86) patients, LDDST was not suppressed but HDDST was suppressed in the combined LDDST and HDDST test.

### Prediction of Intraoperative CSI by Preoperative MRI-Based Knosp Grade

The radiological results represent the first essential step to evaluate CSI of PAs. However, the results of CSI evaluated using Knosp grade based on preoperative MRI are not always consistent with intraoperatively observed CSI. In this study, 1,273 patients were identified as Knosp Grade 0, 1 and 2, and 108 patients were classified into Knosp Grade 3 and 4, respectively, based on preoperative coronal MRI (**Table 2**). None of the Knosp Grade 0 and 1 based on preoperative coronal MRI had shown evidence of intraoperative CSI, and all the MRI-based Knosp Grade 4 patients demonstrated CSI intraoperatively. However, CSI was intraoperatively observed in 5.4% (4 of 74) of patients with MRI-based Knosp Grade 2, and no obvious CSI was found intraoperatively in 16.0% (8 of 50) patients with MRI-based Knosp Grade 3.

**Table 2:** Comparison of results of Knosp grade based on preoperative MRI and surgically observed cavernous sinus invasion.

Knosp grade based on preoperative MRI	Surgically observed invasiveness		Total
	Invasive	Non-invasive	
0 and 1	0	1,199	1,199
2	4	70	74
3	42	8	50
4	58	0	58
Total	104	1,277	1,381

### Surgical Outcome of Different Subgroups

Surgical outcomes of different subgroups based on different variables are shown in **Table 3**. Post-operatively, only 42/86 (48.8%) of overall included patients achieved immediate remission after TSS, whereas 44/86 (51.2%) patients still had persistent disease. Knosp grade was closely related to extent of tumor resection and biochemical remission. In this study, after TSS, only 55.6% (20/36) patients with Knosp Grade 3 and 41.3% (19/46) patients with Knosp grade 4 adenomas achieved biochemical remission, respectively. However, 75% (3/4) of patients with Knosp Grade 2 adenomas achieved remission, which were higher than that in the patients with Knosp grade 3 and 4 adenomas, but did not reach statistical significance (all p > 0.05).

**Table 3:** Rate of immediate remission and recurrence by subgroups based on different variables.

Variable	Number of patients	Remission	Persistence	*p*-value
**Total patients Degree of invasion**	86	42/86 (48.8%)	44/86 (51.2%)	
Knosp Grade 2 (a)	4 (4.7%)	3/4 (75%)	1/4 (25%)	a vs. b: *p* = 0.46
Knosp Grade 3 (b)	36 (41.9%)	20/36 (55.6%)	16/36 (44.4%)	a vs. c: *p* = 0.19
Knosp Grade 4 (c)	46 (53.5%)	19/46 (41.3%)	27/46 (58.7%)	b vs. c: *p* = 0.20
**Extent of tumor resection**				
GTR (d)	63 (73.3%)	38/63 (60.3%)	25/63 (39.7%)	d vs. e: *p* = 0.004
STR (e)	18 (20.9%)	4/18 (22.2%)	14/18 (77.8%)	d vs. f: *p* = 0.008
PR (f)	5 (5.8%)	0/5 (0%)	5/5 (100%)	e vs. f: *p* = 0.25
**Tumor size**				
Microadenomas (g)	28 (32.6%)	17/28 (60.7%)	11/28 (39.3%)	g vs. h: *p* = 0.12
Macroadenomas (h)	54 (62.8%)	23/54 (42.6%)	31/54 (57.4%)	g vs. i: *p* = 0.18
Giant adenomas (i)	4 (4.7%)	1/4 (25.0%)	3/4 (75.0%)	h vs. i: *p* = 0.49
**Approach**				
Microscopic approach (j)	31 (36.0%)	14/31 (45.2%)	17/31 (54.8%)	
Endoscopic approach (k)	55 (64.0%)	28/55 (50.9%)	27/55 (49.1%)	j vs. k: *p* = 0.61
**Number of operations**				
First TSS (l)	66 (76.7%)	38/66 (57.6%)	28/66 (42.4%)	
Repeat TSS (m)	20 (23.3%)	4/20 (20.0%)	16/20 (80.0%)	l vs. m: *p* = 0.003

Extent of tumor resection has been considered one of the most important factors affecting the surgical outcomes. In this study, GTR was achieved in 73.3% (63/86) patients, and remission was obtained in 60.3% (38/63) of these patients. STR was performed in 20.9% (18/86) patients, and 22.2% (4/18) of them achieved remission. PR were performed in 5 (5.8%) patients, and none achieved initial remission. The remission rate in patient performed with GTR was significantly higher than that in the patients with STR and RR (all p < 0.05).

Tumor size may be another important factor correlated with outcomes of surgery. In this study, a total of 86 included patients had 28 (32.6%) microadenomas, 54 (62.8%) macroadenomas, and 4 (4.7%) giant adenomas, and the postoperative remission rate in these patients were 60.7% (17/28), 42.6% (23/54), and 25.0% (1/4), respectively. However, there is no significant deference in remission rate among three groups (all p > 0.05).

TSS can be performed by microscopic or endoscopic approach, whether endoscopic approach yielded higher remission rate over the microscopic approach is still controversial. In this study, 31 patients underwent TSS using a microscopic approach and 55 patients had an endoscopic approach. However, no significant difference in the remission rate was observed between the two techniques in corticotroph adenomas with CSI (*p* = 0.61).

To further analyze the effect of the number of operations on the surgical outcome of corticotroph adenomas with CSI, patients were subclassified into two groups based on the status of first or repeated TSS. In this study, 66 patients underwent first TSS and 20 patients underwent repeated TSS, respectively. In the patients who underwent repeated TSS, only 20.0% (4/20) of them achieved immediate remission, which is significantly lower than the remission rate (57.6%, 38/66) in the patients who underwent first TSS (*p* = 0.003).

### Perioperative Complications

The surgery for corticotroph adenomas with CSI usually was related to the high incidence of complications. In this series, the most common complication was intraoperatively CSF leakage, which occurred in in 24.4% (21/86) patients (**Table 4**). The sellar reconstruction was performed by packing of the sphenoid sinus and sella turcica with fat, muscle, and nasoseptal flap for all 21 patients with intraoperative CSF leakage. However, there were still 5 patients who have postoperative CSF leakage. Of these, 3 patients were successfully treated by repeated sellar reconstruction and external lumbar drain. Two patients who experienced CSF leakage and CNS infection were treated with antibiotics. After TSS, 15.1% (13/86) of the patients experienced transient diabetes insipidus. Of these, 9 of 13 recovered after medical therapy, and 4 patients developed permanent diabetes insipidus and needed lifelong treatment with desmopressin acetate. After surgery, 5.8% (5/86) of the patients experienced hypopituitarism and received hormone replacement therapy. Five patients (5.8%) experienced partial abducent nerve palsy and three patients (3.5%) experienced partial oculomotor nerve palsy, respectively, and all recovered completely within 1 year. Three patients showed visual deterioration after TSS, and two of them experienced various degrees of improvement of visual dysfunction. One patient who was undergoing repeated TSS experienced ICA rupture (which was controlled by direct compression) and underwent digital subtraction angiography immediately. The ICA rupture was blocked by the stent, and the patient recovered finally without neurological deficits.

**Table 4:** Perioperative complications of patients with corticotroph adenomas invading CS based on Knosp grade.

Results	Overall	Knosp Grade 2	Knosp Grade 3	Knosp Grade 4	*p*-value
Intraoperatively CSF leakage	21/86 (24.4%)	0/4 (0%)	8/36 (22.2%)	13/46 (28.3%)	*p* > 0.05
Postoperatively CSF leakage (%)	5/86 (5.8%)	0/4 (0%)	1/36 (2.8%)	4/46 (8.7%)	*p* > 0.05
Transient diabetes insipidus	13 /86 (15.1%)	1/4 (25.0%)	4/36 (11.1%)	8/46 (17.4%)	*p* > 0.05
Permanent diabetes insipidus	4/86 (4.7%)	0/4 (0%)	2/36 (5.6%)	2/46 (4.3%)	*p* > 0.05
Postoperative hypopituitarism	5/86 (5.8%)	1/4 (25.0%)	2/36 (5.6%)	2/46 (4.3%)	*p* > 0.05
Partial abducent nerve palsy (%)	5/86 (5.8%)	0/4 (0%)	2/36 (5.6%)	3/46 (6.5%)	*p* > 0.05
Partial oculomotor nerve palsy (%)	3/86 (3.5%)	0/4 (0%)	2/36 (5.6%)	1/46 (2.2%)	*p* > 0.05
Impaired vision	3/86 (3.5%)	0/4 (0%)	1/36 (2.8%)	2/46 (4.3%)	*p* > 0.05
CNS infection (%)	2/86 (2.3%)	0/4 (0%)	1/36 (2.8%)	1/46 (2.2%)	*p* > 0.05
ICA rupture (%)	1/86 (1.2%)	0/4 (0%)	0/36 (0%)	1/46 (2.2%)	*p* > 0.05

### Recurrence

Recurrence was observed in 16.7% (7/44) of all patients with initial remission, and the median time to recurrence for these patients was 33.0 months (range 2.0–105.0 months) (**Table 5**). However, no patients with Knosp Grade 2 were found to be recurrent. No significant difference in recurrence rate was observed between the patients with Knosp Grade 3 (15.0%, 3/20) and Knosp Grade 4 adenomas (21.1%, 4/19) (p > 0.05).

**Table 5:** Recurrence outcome of included patients with initial remission based on Knosp grade.

MRI results	Overall	Knosp Grade 2 0/3 (0%)	Knosp Grade 3 3/20 (15.0%)	Knosp Grade 4 4/19 (21.1%)	*p*-value
Number of patients	7/42 (16.7%)				*p* = 0.62
Median time to recurrence (ms)	33.0 (2.00–105.0)	0	29.0 (6.0–56.0)	36.0 (2.0–105.0)	*p* > 0.05

### Further Treatments for Persistent and Recurrent Corticotroph Adenomas With CSI

For those patients with persistent disease (44) and recurrent corticotroph adenomas (7), data about further treatment were available for only 30 of 51 patients. Radiotherapy was used for 15 patients, and 4 (26.7%) of them achieved biochemical remission. Repeat TSS was performed in 5 patients, but none achieved remission. Medication was administered in 4 patients, and only one obtained disease control. Adrenalectomy was performed in 6 patients, and 5 (83.3%) achieved biochemical remission. At the last follow-up, 33.3% (10 of 30) of the patients were in remission, and 20 patients still had persistent disease (**Table 6**).

**Table 6:** Further treatments and remission rate in persistent and recurrent patients with invasive corticotroph adenomas.

Total/Remission Rate	Radiotherapy	Repeat TSS	Medical therapy	Adrenalectomy
Total (10/30, 33.3%)	4/15 (26.7%)	0/5 (0%)	1/4 (25.0%)	5/6 (83.3%)

## Discussion

This study reports on the outcomes of TSS in 86 patients with corticotroph adenoma invading CS who underwent TSS at the PUMCH between 2000 and 2019. To our knowledge, this is one of the largest series on corticotroph adenomas with CSI published till now. This study demonstrated that the immediate remission of the surgical approach and the long-term outcome after adjuvant therapies for corticotroph adenomas with CSI are unsatisfactory. Additionally, CSI evaluated using Knosp grade based on preoperative MRI not always accurately predict intraoperatively observed CSI. Moreover, extent of tumor resection and number of operations significantly affected the surgical outcome of TSS for corticotroph adenomas with CSI. However, the Knosp grade, tumor size, and technical factor is not associated with the surgical outcome.

Preoperative MRI-based Knosp grade classifications play a crucial role in diagnosis of CSI of corticotroph adenomas. However, radiological results do not always discriminate between compression/extension and invasion of CS. In this study, only 84.0% (42 of 50) of the patients with MRI-based Knosp grade 3 was intraoperatively identified with CSI, and 8 other patients with Knosp grade 3 adenomas did not have CSI at surgery. Furthermore, 5.4% (4 of 74) of the patients with MRI-based Knosp grade 2 adenomas were found to have CSI during surgery. These findings are consistent with previous studies (15, 16). Dickerman and colleagues (17) demonstrated that dural invasion was directly observed at surgery and was confirmed histologically in 62% of the patients with no adenomas and interpreted based on preoperative MRI. Lonser also reported that preoperative MRI accurately predicted dural invasion in only 4 patients (22%) with CSI (18). Other previous studies also indicated that Knosp classification does not accurately predict the invasion of the Knosp grade 0–2 adenomas; thus, their application in the prediction of invasion among microadenomas is limited (19). A recent Meta-Analysis also reported that the prevalence of CSI radiographically (43%) was much higher than that (18%) intraoperatively, and the radiologic criteria of Knosp 3-4 had the highest correlation with intraoperative CSI (20). Therefore, although MRI-based Knosp grade can reliably define the degrees of CSI in Knosp 3–4 larger tumors, it is often unreliable to define the absence of CSI in Knosp 0–2 microadenomas. To define more accurately CSI beyond the lateral tangential line between ICA segments respectively, Knosp updated the original grading system of invasion in PAs by establishing the subtypes of grade 3a and 3b PAs in 2015 (15). However, up to 80% of CD patients present with a microadenoma, and there is no reliable grading system for microadenomas that accurately predicts CSI of corticotroph adenoma. Thus, more reliable grading systems of invasion for corticotroph adenomas are needed.

For corticotroph adenomas with CSI, complete surgical resection of tumor is difficult. Thus, lower remission rates of TSS have been reported in the patients with corticotroph adenomas with CSI. However, 48.8% of corticotroph adenomas with CSI in our center achieved remission after TSS, which was higher than that reported in previous studies (7, 21). The main reasons for the higher remission rate may include the experienced neurosurgeons, intraoperative multiple technique assistance, and aggressive surgical procedure.

Surgery for corticotroph adenomas with CSI has always been a challenge because of the highly complex anatomy of the CS and difficult in CS dissection. Thus, experienced neurosurgeon is essential to achieve complete tumor resection, biochemical remission, and avoid perioperative complications. In our center, almost all surgeries for CD patients were performed by RW and MF, who had experience with more than one thousand pituitary surgeries as previously described (9). Each year, more than one hundred CD patients undergo pituitary surgery in PUMCH, and a large part of them are invasive macroadenomas and recurrent CD (22). The number of surgical patients with CD in PUMCH may be one of the largest centers. Therefore, large-scale surgical patients with CD have accumulated rich surgical experience in management of patients with corticotroph adenomas with CSI. Unfortunately, even in the hands of experienced surgeons, only about half of corticotroph adenomas with CSI could achieve remission after TSS. There are several studies that also found that biochemical remission rate is related to the number of years of neurosurgical experience. Yap and colleagues reported that the first decade of neurosurgery experience was associated with lower remission rates than that with the second and third decade of neurosurgery experience (23). A recent meta-analysis also demonstrated the possible association of neurosurgeons’ experience with remission rates in CD patients (24). Therefore, neurosurgical experience may be one of main reasons for the higher remission rate of TSS in corticotroph adenomas with CSI.

Application of multiple techniques may be another important factor for the higher remission rate of TSS in corticotroph adenomas with CSI. In this study, multiple techniques including neuronavigation and intraoperative Doppler ultrasonography were used intraoperatively for surgical assistance in most patients, which resulted in maximum tumor removal and a relatively low rate of perioperative complications. For larger and recurrent corticotroph adenomas with CSI, neuronavigation and Doppler ultrasonography was used to determine the exact location of the ICA. These techniques can provide references for locating some important structures including the ICA, brainstem, and optic canal in real time during surgery (25). Precisely locating these structures can prevent injuries to them, thereby decreasing the frequency of perioperative complications. T. J. Owen also reported that using the neuronavigation system for localization, the rostral and caudal margins of the pituitary fossa during TSS may decrease morbidity and surgical time (26). Doppler ultrasonography has also been shown to determine the exact location of the ICA and whether an aneurysm exists, thereby avoiding injuring the ICA during surgery (27). Therefore, these multiple techniques used in surgery facilitate tumor resection and a safe operation and decrease perioperative complications in corticotroph adenomas with CSI.

In our center, an aggressive procedure was used to pursue a maximum safe removal of tumor. During the surgery, in order to accurately assess the CSI and to remove the tumor maximally, it is critical to widely expose the anterior and inferior sella dura, even the medial dural wall of the CS. Thus, endoscopic extended transsphenoidal surgery was used for most larger corticotroph adenomas with CSI, which provide direct visualization for resection of the tumors invading the CS and suprasellar (28). Recently, the incision of the CS wall has also been performed for PAs invading the CS, which resulted in a higher GTR rate (12). Therefore, rich surgical experience, wide exposure by endoscopic extended transsphenoidal surgery and incision of the CS, and intraoperative assistance of combined neuronavigation and intraoperative Doppler ultrasonography are the main reasons for the higher remission rate in our center.

Identification of the factors affecting surgical outcomes is very important for predicting the prognosis of patients with corticotroph adenomas invading CS. In this study, we found that the remission rate (75%) in patients with Knosp grade 2 adenomas is higher than that in patients with Knosp grade 3 adenomas (55.6%) and Knosp grade 4 adenomas (41.3%), but it did not reach statistical significance. This result indicated that the MRI-based Knosp grade classifications were not related to immediate remission in corticotroph adenomas with CSI, which is consistent with the results of most previous studies. Similarly, Wagenmakers (6) reported that the remission was achieved in 50.0% of patients with Knosp grade 2, 37.5% of patients with Knosp grade 3, and 33.3% of patients with Knosp grade 4, respectively. However, another study by Witek showed that the immediate postoperative remission depended on invasiveness based on Knosp grades 3 and 4 for macroadenomas (29). However, only 4 patients with Knosp grade 2 adenomas were included in the present study, and more large-scale studies are needed to further verify this conclusion.

Whether the adenoma size affects the surgical remission in corticotroph adenomas with CSI remains controversial. In our study, patients with microadenomas invading CS had higher immediate remission rates than patients with macroadenomas invading CS and giant adenomas invading CS; however, it does not reach statistical significance. This is consistent with the results from some previous studies. Starke reported that there was no significant difference in remission among patients with microadenomas and macroadenomas (30). Feng also reported that CD patients with macroadenomas and microadenomas had similar remission rate after TSS (11). On the contrary, a few studies have opposite conclusions; Blevins (31) indicated that CSI and the presence of a tumor diameter ≥ 2.0 cm were characteristics associated with an increased likelihood of residual disease after surgery. Other studies also demonstrated an inverse correlation between remission rates and tumor size in patients with CD (4, 32). However, most of the previous studies reported the effect of tumor diameter on remission rate in patients with CD, but not in patients with corticotroph adenoma invading CS. Therefore, more studies on potential factors predicting surgical outcomes in corticotroph adenomas with CSI are needed.

In this study, we found that the repeated TSS for corticotroph adenomas with CSI has shown significant lower remission rates compared with the first TSS. The main reasons for this result may be the destruction of the original anatomy and scar formation within the recurrent tumor. These factors make the surgery more difficult and dangerous. Valderrábano and colleagues (33) also demonstrated that the repeat TSS for CD is associated to a lower remission rate and a higher risk of recurrence, which is consistent with our results. However, the large-scale clinical study on results of repeat TSS for corticotroph adenomas with CSI is limited, and further studies are needed.

Depending on the neurosurgeon’s preference, TSS for corticotroph adenomas with CSI could be performed using a microscopic or endoscopic approach. Compared with the microscopic approach, the endoscopic approach provides a broader surgical view of the pituitary region, including lateral edges of the sella and CS. However, in this study, no significant difference was found in the remission rates among the patients with corticotroph adenomas invading CS who underwent microscopic TSS and endoscopic TSS. The microscopic and endoscopic techniques were used in combination for a subset of corticotroph adenomas with CSI, which might explain why an endoscopic versus a microscopic technique yielded a similar remission rate in our center. This result is in accordance with one recent meta-analysis, which indicated that comparisons of remission rates by endoscopic versus microscopic technique yielded the same results (34). However, another meta-analysis demonstrated that the endoscopic TSS reaches comparable results for microadenomas, and probably better results for macroadenomas than microscopic TSS for CD patients (35). To date, the data about comparisons of remission rates by an endoscopic versus a microscopic technique in corticotroph adenomas with CSI are limited, and more studies comparing the two techniques in corticotroph adenomas with CSI at the same institution are needed.

We have previously indicated that the number of operations, the duration of disease, tumor invasion, tumor size, and preoperative ACTH concentration could predict the immediate outcomes of TSS in patients with corticotroph adenomas (36). However, in this study, we found that neither Knosp grade nor tumor size affects the remission rate in corticotroph adenomas with CSI. The main reasons for the difference may be that all included corticotroph adenomas are invading CS in this study; therefore, the Knosp grade and tumor size did not affect the surgical outcomes. Compared with the first TSS, the repeated TSS for corticotroph adenomas with CSI is related to the lower remission rates, which is consistent with the results of previous studies (37, 38). Therefore, further research is needed to identify the factors affecting surgical outcomes of corticotroph adenomas with CSI.

It is difficult to manage patients with persistent or recurrent corticotroph adenomas with CSI. Therapy options include radiation therapy, repeated TSS, medical therapy, and, as a final step, bilateral adrenalectomy. These treatments have their own advantages and disadvantages; however, there is no consensus on which treatment is preferable. In our center, an individual-based comprehensive treatment was discussed by a multidisciplinary team (MDT) with collaborating experts. However, even if comprehensive treatments were used, the prognosis of these patients is still poor, and more effective treatments are needed.

## Limitations

Although this study is a rather larger patient cohort regarding the results of TSS on corticotroph adenomas with CSI in a single institution, there are some limitations that deserve to be mentioned. First, because of the retrospective nature of the study, it is difficult to collect complete clinical information and long-term follow-up data for all patients. Some data on long-term follow-up and further treatments were missing, which may result in a bias of the results and conclusions on long-term outcomes and recurrence rate. Additional information of survival or death for half of the included patients cannot be obtained; thereby, the mortality results are not included and analyzed. Another limitation is that referral or selection bias must be taken into consideration because this was a single-center cohort study.

## Conclusions

In summary, the remission rate of corticotroph adenomas with CSI was unsatisfactory due to incomplete resection of invasive and/or a large adenoma. With the application of multiple techniques, approximately half of the patients could achieve GTR and biochemical remission *via* TSS by experienced neurosurgeons. Extent of tumor resection and number of operations were associated with surgical remission rate in corticotroph adenomas with CSI. In contrast, Knosp grade, tumor size, and surgical approach did not affect the remission rate of TSS in corticotroph adenomas with CSI. In addition, further treatment including radiotherapy, repeated operation, medical therapy, and even bilateral adrenalectomy are required for persistent or recurrent corticotroph adenomas with CSI.

## Data Availability Statement

The raw data supporting the conclusions of this article will be made available by the authors, without undue reservation.

## Ethics Statement

The studies involving human participants were reviewed and approved by the Ethics Committee of Peking Union Medical College Hospital (Reference number: S-K1423). Written informed consent to participate in this study was provided by the participants’ legal guardian/next of kin.

## Author Contributions

All authors listed have made a substantial, direct, and intellectual contribution to the work, and approved it for publication.

## Funding

Financial support for this study was provided by the Beijing Municipal Administration of Hospitals Incubating Program (grant number: PX2022004). The funding institutions had no role in the design of the study, data collection and analysis, decision to publish, or preparation of the manuscript.

## Conflict of Interest

The authors declare that the research was conducted in the absence of any commercial or financial relationships that could be construed as a potential conflict of interest.

## Publisher’s Note

All claims expressed in this article are solely those of the authors and do not necessarily represent those of their affiliated organizations, or those of the publisher, the editors and the reviewers. Any product that may be evaluated in this article, or claim that may be made by its manufacturer, is not guaranteed or endorsed by the publisher.
